# Ginger Polyphenols Reverse Molecular Signature of Amygdala Neuroimmune Signaling and Modulate Microbiome in Male Rats with Neuropathic Pain: Evidence for Microbiota–Gut–Brain Axis

**DOI:** 10.3390/antiox13050502

**Published:** 2024-04-23

**Authors:** Chwan-Li Shen, Julianna Maria Santos, Moamen M. Elmassry, Viren Bhakta, Zarek Driver, Guangchen Ji, Vadim Yakhnitsa, Takaki Kiritoshi, Jacob Lovett, Abdul Naji Hamood, Shengmin Sang, Volker Neugebauer

**Affiliations:** 1 Department of Pathology, Texas Tech University Health Sciences Center, Lubbock, TX 79430, USA; julianna.santos@ttuhsc.edu (J.M.S.);; 2 Center of Excellence for Integrative Health, Texas Tech University Health Sciences Center, Lubbock, TX 79430, USA; volker.neugebauer@ttuhsc.edu; 3 Center of Excellence for Translational Neuroscience and Therapeutics, Texas Tech University Health Sciences Center, Lubbock, TX 79430, USA; 4 Department of Molecular Biology, Princeton University, Princeton, NJ 08540, USA; 5 Department of Biology, Texas Tech University, Lubbock, TX 79401, USA; 6 Department of Biochemistry, Texas Tech University, Lubbock, TX 79401, USA; 7 Department of Pharmacology & Neuroscience, Texas Tech University Health Sciences Center, Lubbock, TX 79430, USA; guangchen.ji@ttuhsc.edu (G.J.); vadim.yakhnitsa@ttuhsc.edu (V.Y.); takaki.kiritoshi@ttuhsc.edu (T.K.); 8 Department of Microbiology and Infectious Disease, Texas Tech University Health Sciences Center, Lubbock, TX 79430, USA; abdul.hamood@ttuhsc.edu; 9 Laboratory for Functional Foods and Human Health, Center for Excellence in Post Harvest Technologies, North Carolina A&T State University, North Carolina Research Campus, Kannapolis, NC 28081, USA; ssang@ncat.edu

**Keywords:** ginger, pain, gut microbiome, neuroinflammation, brain

## Abstract

Emerging evidence shows that the gut microbiota plays an important role in neuropathic pain (NP) via the gut–brain axis. Male rats were divided into sham, spinal nerve ligation (SNL), SNL + 200 mg GEG/kg BW (GEG200), and SNL + 600 mg GEG/kg BW (GEG600) for 5 weeks. The dosages of 200 and 600 mg GEG/kg BW for rats correspond to 45 g and 135 g raw ginger for human daily consumption, respectively. Both GEG groups mitigated SNL-induced NP behavior. GEG-supplemented animals had a decreased abundance of *Rikenella*, *Muribaculaceae*, *Clostridia UCG-014*, *Mucispirillum schaedleri*, *RF39*, *Acetatifactor*, and *Clostridia UCG-009*, while they had an increased abundance of *Flavonifactor*, *Hungatella*, *Anaerofustis stercorihominis*, and *Clostridium innocuum* group. Relative to sham rats, *Fos* and *Gadd45g* genes were upregulated, while *Igf1*, *Ccl2*, *Hadc2*, *Rtn4rl1*, *Nfkb2*, *Gpr84*, *Pik3cg*, and *Abcc8* genes were downregulated in SNL rats. Compared to the SNL group, the GEG200 group and GEG600 group had increases/decreases in 16 (10/6) genes and 11 (1/10) genes, respectively. GEG downregulated *Fos* and *Gadd45g* genes and upregulated *Hdac2* genes in the amygdala. In summary, GEG alleviates NP by modulating the gut microbiome and reversing a molecular neuroimmune signature.

## 1. Introduction

Neuropathic pain (NP) arises from damage to the peripheral or central nervous system (CNS) [1]. Nerve damage in NP leads to neuroinflammation and neuroplastic changes in the peripheral and central nervous systems (CNS) associated with sensitization and hyperexcitability [2]. The challenges of chronic NP are related to the complexity of NP symptoms (e.g., anxiety and depression), poor outcomes, and limited availability of treatment options. The most-used form of NP treatment is opioid analgesics; unfortunately, they can induce severe side effects and result in opioid use disorder [3].

Accumulating evidence suggests that the gut microbiome has a great impact on NP as an important element of the gut–brain axis via modulating neuroinflammation [4]. Gut dysbiosis has been implicated in the onset or progression of NP-associated behavior, such as pain sensitivity [5,6,7,8]. Gut dysbiosis is not only associated with marked changes in gut-associated immune cell activation in lymphoid tissues; it also exacerbates spinal inflammation/lesions, leading to impaired recovery of neurological function [9,10]. A recent systematic review with 19 eligible studies provides a rationale for targeting the microbiota for managing symptoms of neuropathy and neuroinflammation-based CNS disorders [11].

NP mechanisms include an imbalance between endogenous antioxidants and reactive oxygen species (ROS) after nerve injury, resulting in neuroimmune cross-talk [12] and neuroinflammation in the peripheral and CNS [13]. Therefore, the advancement of new, safe, and effective analgesic and anti-inflammatory alternatives is keenly desired. Ginger (*Zingiber officinale* Roscoe) consists of gingerols (6-gingerol, 8-gingerol, and 10-gingerol) and shogaols (6-shogaol, 8-shogaol, and 10-shogaol) that account for its anti-inflammatory properties [14]. Myriad ginger extract and its bioactive compounds have been investigated as anti-inflammatory agents; the length of their side chains influences their effectiveness [15]. Ginger and its bioactive components have been demonstrated to penetrate the blood–brain barrier via passive diffusion, suggesting the positive effects of ginger on the CNS [16].

Our previous work linked ginger’s anti-inflammatory and antioxidant properties to antinociception [17,18]. Single-dosage gingerol-enriched ginger (GEG) dietary supplementation significantly mitigated mechanical hypersensitivity in rats with spinal nerve ligation (SNL)-induced NP via, in part, (i) modulation of the gut microbiota and metabolites [17] and (ii) suppression of mRNA *NF-κB* and *TNF-α* expression in the amygdala and colon [18]. Here, we explored the effects of GEG on neuroimmune signaling with a focus on the amygdala for the following reasons. The amygdala has emerged as a key brain area for pain modulation and the affective component of pain [19], which, according to the International Association for the Study of Pain (IASP), is what defines pain [20]. Neuroplasticity in the amygdala has been linked to pain behaviors. While most research on underlying mechanisms has focused on neuronal mechanisms such as synaptic plasticity and hyperexcitability, recent evidence suggests that neuroimmune signaling contributes to pain mechanisms in the amygdala [18,21]. Finally, the amygdala has been recognized as a key region and hub for brain–gut interactions [22,23].

In the current study, we further investigated how two dosages of GEG administration via oral gavage affect 770 neuroinflammatory signature genes in the amygdala of SNL-treated animals, using the NanoString neuroinflammation panel. These neuroinflammation panels are designed to swiftly analyze important aspects of neuroimmune interactions for a thorough perspective of the complex relationship between immune and nervous systems. This neuroinflammation panel includes 23 pathways and processes that represent three core themes of neuroinflammation: stress and metabolism, immunity and inflammation, and neuropathology and neurobiology. In addition, we also conducted pain assessment and gut microbiome analysis in the cecal feces of the animals. Different from previous studies where GEG was delivered via diet at a single dosage [17,18], in the present study, GEG was given via oral gavage at two dosages (200 mg/kg and 600 mg/kg body weight daily) to evaluate if there was any response in the outcome parameters, namely, mechanical hypersensitivity, gut microbiome composition, and neuroinflammation signature gene expression.

We hypothesized that GEG administration would reduce mechanical hypersensitivity in a GEG-dose-dependent manner. Such changes in mechanical hypersensitivity would be mediated by (i) modification of the gene expression of three core themes of neuroinflammation and (ii) modulation of the gut microbiome composition with a greater abundance of beneficial microorganisms due to GEG administration. In this study, we combined a comprehensive evaluation of a neuroinflammation panel and gut microbiome abundance/composition to better understand the effects of ginger’s bioactive compounds on metabolic pathways relevant to NP in the development of personalized nutrition therapy for NP management.

## 2. Materials and Methods

### 2.1. Animals

Thirty-six male Sprague Dawley rats (4–5 weeks old, 150–180 g, Envigo, Cumberland, VA, USA) were individually housed under a 12 h light–dark cycle. All animals were given access to food and water ad libitum throughout the study period. All experimental procedures were approved by the Institutional Animal Care and Use Committee at Texas Tech University Health Sciences Center (IACUC #20032). Food consumption, water intake, and body weights were recorded weekly.

### 2.2. Neuropathic Pain Induction

We employed a spinal nerve ligation (SNL) preclinical model to study the effects of ginger extract on NP progression. SNL leads to acute hypersensitivity within 1 week that persists for weeks [24] and prolonged changes in inflammatory and pronociceptive mediators, neurotransmitters, and receptor expression, leading to peripheral and central sensitization [25]. The SNL model was used to induce peripheral neuropathy in the left hind paw [2,26].

After a 5-day acclimatization, 9 animals received the sham procedure, while the remaining 27 animals received the SNL procedure. We used isoflurane [induction (3%) and maintenance (2%) of anesthesia] throughout the sham or SNL procedure. After removing the L5/L6-level paraspinal muscles and the underlying L6 transverse process, the L5 spinal nerve was separated from adjacent structures and tightly ligated with 6-0 silk thread. The paraspinal muscles were sutured closed, and the skin clipped together. Sham-operated animals served as controls for the NP model, receiving the same surgical procedure without the L5 spinal nerve ligation. After surgery, we applied ointment antibiotics (VetOne, Boise, ID, USA) to the surgery site until the staples were removed. Throughout the study period, we monitored the animals to reduce unnecessary stress or pain following the ethical guidelines of the International Association for the Study of Pain [27].

### 2.3. Animal Treatments

Thirty-six animals were randomly assigned into the sham + vehicle (corn oil) group (the sham group), SNL + vehicle (corn oil) group (the SNL group), SNL + 200 mg GEG/kg BW group (the SNL + GEG200 group), and SNL + 600 mg GEG/kg BW group (the SNL + GEG600 group). Both corn oil (vehicle) and GEG were administered by oral gavage for 4 weeks. All animals were given an AIN-93G diet (catalog number # D10012G, Research Diet, Inc., New Brunswick, NJ, USA). Prior studies that administered ginger extract to rats (concentrations ranging between 100 mg and 400 mg/kg BW) showed a decrease in inflammation in rats [28,29]. Thus, in this study, we tested GEG at both 200 and 600 mg/kg BW dosages in an NP model.

Ginger (*Zingiber officinale*) rhizomes were harvested, cleaned with water, and dried in the shade. Once dried, the ginger rhizomes were pulverized to a coarse powder form. The powdered ginger was subjected to supercritical fluid extraction to obtain a soft ginger extract standardized to 20% gingerols. Based on the results of gas chromatography–mass spectrometry, GEG consists of 18.7% 6-gingerol, 1.81% 8-gingerol, 2.86% 10-gingerol, 3.09% 6-shogoal, 0.39% 8-shogaol, and 0.41% 10-shogaol. GEG was a gift obtained from Sabinsa, Inc., East Windsor, NJ, USA.

### 2.4. Assessment of Pain-Related Behavior in Live Animals

We used the von Frey test to measure mechanical hypersensitivity [2]. In brief, we measured mechanical paw withdrawal thresholds (in grams) using an Electronic von Frey Aesthesiometer (IITC Life Science, Woodland Hills, CA, USA) with a plastic tip in an exclusive testing area for pain sensory assessment. The average of six measurements at least 30 s apart was calculated for each animal test.

### 2.5. Sample Collection

On collection day (after a 5-week feeding period), the animals were anesthetized and euthanized, and blood was drawn for plasma collection. The amygdala (right) and cecal feces were harvested, immersed in liquid nitrogen, and kept at −80 °C. We focused on the right amygdala because of previous evidence for the right hemispheric lateralization of pain plasticity and pain modulation in the amygdala [30,31,32].

### 2.6. RNA Isolation and Gene Expression Profiling Using Neuroinflammation Panel

We extracted total RNA from the amygdala using a Qiagen RNeasy Mini Kit (Cat # 74106, Qiagen Science, Germantown, MD, USA). We measured the purity/concentration of RNA using Nanodrop 2000 (Thermo Fisher Scientific, Waltham, MA, USA) and stored RNA at −80 °C. We shipped amygdala RNA samples (20 ng/µL) to Cleveland Clinic, Cleveland, OH, USA, to perform gene expression profiling using the nCounter^®^ neuroinflammation pathway panel (NanoString Technologies, Seattle, WA, USA). The neuroinflammation panel includes 757 genes covering the core pathways and processes that define neuroinflammation interactions and 13 potential housekeeping genes for normalization. RNA samples (100 ng per sample) were used for the Gene Expression Assay with a neuroinflammation panel using the nCounter MAX system, a multi-channel epifluorescence scanner with the NanoString Advanced Analysis Module plugin for quality control. We analyzed raw datasets using the ROSALIND^®^ platform (https://rosalind.bio/ (accessed on 9 December 2022)). Sample gene transcript counts were normalized by dividing counts within a lane by the geometric mean of the normalizer probes from the same lane. Housekeeping probes for normalization were selected based on the geNorm algorithm using the NormqPCR R package (version 1.48) [33]. The abundance of various cell populations was calculated using the Nanostring Cell Type Profiling Module within ROSALIND. ROSALIND performs a filtering of Cell Type Profiling results to include results that have scores with a *p*-value greater than or equal to 0.05. Hypergeometric distribution was used to analyze the enrichment of pathways, gene ontology, domain structure, and other ontologies. NanoString annotation term enrichment was calculated relative to a set of background genes relevant to the experiment. Visualization was performed in R version 4.0.5 (Shake and Throw).

### 2.7. RNA Isolation and qRT-PCR

We validated the results of neuroinflammation gene profiling [FOS (Fos proto-oncogene-encoding proteins that form the AP-1 transcription factor complex), Gadd45g (growth arrest and DNA-damage-inducible 45 gamma), and HDAC2 (histone deacetylase 2)] using qRT-PCR. Extracted RNA was reversely transcribed into cDNA using the Maxima first-strand cDNA synthesis kit synthesis with dsDNase (Thermo Scientific, K1672, Waltham, MA, USA) on a thermal cycler Bio-rad S1000 (Bio-Rad Laboratories, Inc., Hercules, CA, USA). qRT-PCR was performed on the Quant Studio 12K Flex real-time PCR system (Life Technologies, 4470689, Carlsbad, CA, USA) using samples cDNA for the amplification of target genes with β-actin as the control with Universal SYBR green supermix (Bio-rad Laboratories, Inc., 17251-24, Hercules, CA, USA). The following genes were tested: inflammation markers (*FOS*, *Gadd45g*, and *HDAC2*). The primer sequences used are below in Table 1. All gene expressions were normalized to our control β-actin. Gene expression was calculated by the following formula: 2-(ΔCT*1000) [34].

### 2.8. Gut Microbiota Profiling via 16S rRNA Amplicon Sequencing

Fecal DNA was isolated using the PowerFecal DNA isolation kit (Qiagen Inc., Germantown, MD, USA). Amplicon sequencing of the V4 variable region of the 16S rRNA gene was conducted at Molecular Research LP (Shallowater, TX, USA). Briefly, the V4 variable region was amplified using PCR primers 515F/806R. Samples were multiplexed and pooled together in equal proportions based on their molecular weight and DNA concentrations. Pooled samples were purified using calibrated Ampure XP beads, and then used in Illumina DNA library preparation. Sequencing was performed on a MiSeq. We deposited raw sequencing data under BioProject accession number PRJNA935472 in the National Center for Biotechnology Information (NCBI) BioProject database. The 16S rRNA gene sequencing data were analyzed using QIIME 2 [35]. In brief, reads were filtered, denoised, and merged. DADA2 was used to identify exact amplicon sequence variants (ASVs). For the taxonomy assignment, the Silva release 138 database was used [36,37]. To compare the relative abundance of taxa between groups, we performed compositional analysis using LOCOM, a logistic regression model for testing differential abundance in compositional microbiome data with false discovery rate control [38]. Results were regarded as significant when the *p*-value < 0.05, unless stated otherwise. Visualization was performed in R version 4.0.5 (codename “Shake and Throw”).

### 2.9. Statistical Analysis

The data were analyzed by a one-way ANOVA or two-way ANOVA (repeated measures where appropriate) followed by Tukey’s post hoc test using GraphPad Prism software version 9.0 (GraphPad Software, San Diego, CA, USA). The data were checked for normality (Gaussian distribution) before employing ANOVA. A significance level of *p*-value < 0.05 applies to all statistical tests. Statistical analyses for other types of data are stated in their corresponding sections above. For gut microbiota analysis, three pairwise comparisons are described as follows: SNL vs. sham, SNL + GEG200 vs. SNL, and SNL + GEG600 vs. SNL. All comparisons “Group 1 vs. Group 2” should be interpreted as “Group 1 relative to Group 2” in the text and figures.

## 3. Results

### 3.1. GEG Alleviates Mechanical Hypersensitivity in NP Rats

**Figure 1:**
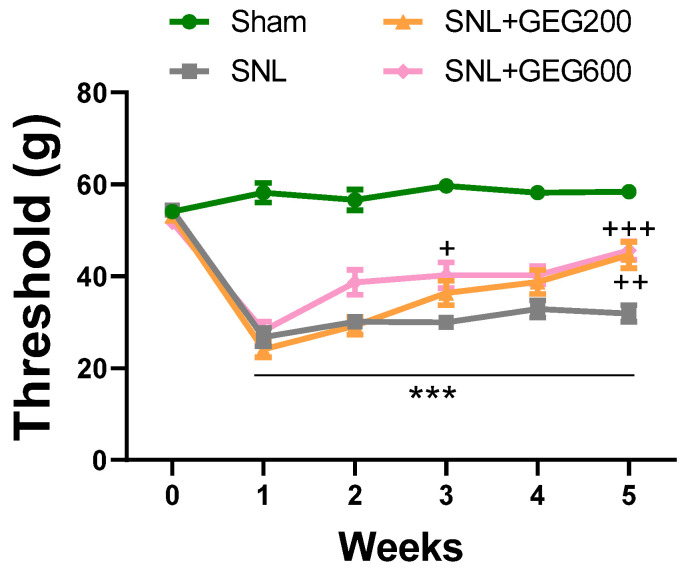
Effects of GEG on mechanical hypersensitivity. Mechanical hypersensitivity of the left hind paw was assessed by an electronic von Frey anesthesiometer. Data expressed as mean ± SEM. n = 9 per group. SNL rats showed significantly decreased mechanical thresholds, indicating that SNL surgery induced pain-related hypersensitivity (*** *p* < 0.001 compared with sham, two-way ANOVA with Tukey’s multiple comparisons test, n = 9). SNL + GEG200-group rats and SNL + GEG600 group rats showed significantly increased mechanical thresholds (+ *p* < 0.05, ++ *p* < 0.01, +++ *p* < 0.001 compared with SNL, two-way ANOVA with Tukey’s multiple comparisons test, n = 9), indicating that GEG200 or GEG600 application reduced pain-related hypersensitivity.

Figure 1 shows the effects of GEG supplementation on NP-associated mechanical hypersensitivity using the von Frey test. Relative to the sham group, the SNL group had significantly greater mechanical hypersensitivity at 1 week post-operation, which was sustained throughout the study period (Figure 1). At the end of the study (4 weeks after GEG supplementation began), both GEG SNL groups showed significantly attenuated pain sensitivity compared to untreated SNL rats, regardless of GEG dosages (Figure 1).

### 3.2. GEG Reverses the Expression of Neuroinflammatory Markers Associated with NP

**Figure 2:**
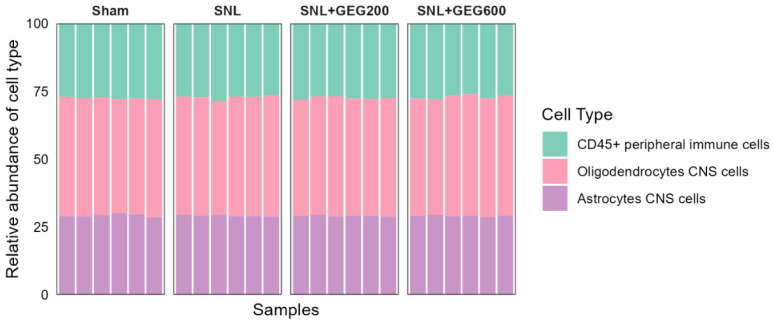
Cell type profiles across groups per NanoString Neuroinflammation Panel analyzed using the ROSALIND platform. The y-axis displays the relative abundance of each cell type, while the x-axis represents samples of different groups. The colors of the bars indicate the different cell types.

We examined the cell type, gene expression, and pathways involved in neuroinflammation and GEG effects and focused on the amygdala because it plays an important role in pain modulation [19]. To achieve this, we used the NanoString nCounter^®^ Neuroinflammation Panel (NanoString Technologies, Inc., Seattle, WA, USA) to profile changes. One of the key outputs of the NanoString nCounter^®^ Neuroinflammation Panel is to measure the relative abundance of 5 CNS cell types and 14 peripheral immune cell types with the unique cell-profiling feature. Based on the analysis performed within ROSALIND, three neuroinflammation cell types were identified, namely CD45^+^ peripheral immune cells, oligodendrocyte CNS cells, and astrocyte CNS cells (Figure 2). In general, oligodendrocytes CNS cells were the most abundant cell type (~50%) across all samples. However, the relative abundance of all three cell types was comparable between groups (ANOVA, *p* > 0.05).

**Figure 3:**
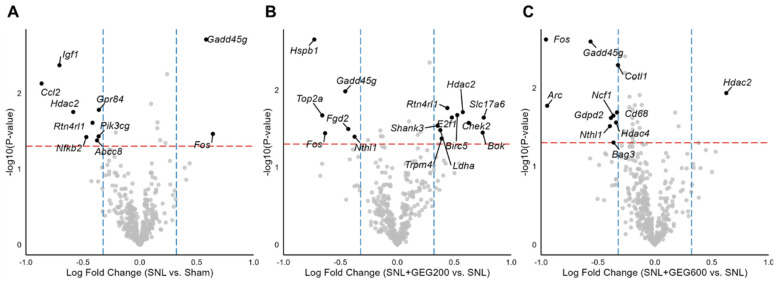
Differential expression of neuroinflammation-related genes between groups. Volcano plots of (**A**) SNL vs. sham, (**B**) SNL + GEG200 vs. SNL, and (**C**) SNL + GEG600 vs. SNL show −log10(*p*-value) on the y-axis and log 2-fold change in gene expression on the x-axis. Statistical significance cutoffs are indicated by a red horizontal dashed line at a *p*-value of 0.05 and blue vertical dashed lines at a fold change of 1.25. Abbreviations: *Abcc8*, ATP-binding cassette subfamily C member 8; *Arc*, activity-regulated cytoskeleton-associated protein; *Bag3*, BAG cochaperone 3; *Birc5*, baculoviral IAP repeat-containing 5; *Bok*, BCL2 family apoptosis regulator; *Ccl2*, C-C motif chemokine ligand 2; *Cd68*, CD68 molecule; *Chek2*, checkpoint kinase 2; *Cotl1*, coactosin-like F-actin-binding protein 1; *E2f1*, E2F transcription factor 1; *Fgd2*, FYVE, RhoGEF and PH domain-containing 2; *Fos*, FBJ osteosarcoma oncogene; *Gadd45g*, growth arrest and DNA-damage-inducible 45 gamma; *Gdpd2*, glycerophosphodiester phosphodiesterase domain-containing 2; *Gpr84*, G protein-coupled receptor 84; *Hdac2*, histone deacetylase 2; *Hdac4*, histone deacetylase 4; *Hspb1*, heat shock protein family B (small) member 1; *Igf1*, insulin-like growth factor-I; *Ldha*, lactate dehydrogenase A; *ncf1*, neutrophil cytosolic factor 1; *Nfkb2*, nuclear factor kappa B subunit 2; *Nthl1*, nth-like DNA glycosylase 1; *Pik3cg*, phosphatidylinositol-4,5-bisphosphate 3-kinase catalytic subunit gamma; *Rtn4rl1*, reticulon 4 receptor-like 1; *Shank3*, SH3 and multiple ankyrin repeat domains 3; *Slc17a6*, solute carrier family 17 member 6; *Top2a*, DNA topoisomerase II alpha; *Trpm4*, transient receptor potential cation channel subfamily M member 4.

Among the 770 gene expressions measured, many differentially expressed genes were identified in each comparison, i.e., SNL vs. sham, SNL + GEG200 vs. SNL, and SNL + GEG600 vs. SNL in volcano plots (Figure 3A–C) (fold change > 1.25 and *p* < 0.05). Ten genes (two increased and eight decreased) were differentially expressed in the amygdala of rats between the SNL and sham groups (Figure 3A). Compared to the sham group, the SNL group had higher gene expression levels of *Gadd45g* (growth arrest and DNA-damage-inducible 45 gamma) and *Fos* (FBJ osteosarcoma oncogene), while it had lower gene expression levels of *Igf1*, *Ccl2*, *Hdac2*, *Rtn4rl1*, *Nfkb2*, *Gpr84*, *Pik3cg*, and *Abcc8* (Figure 3A).

After identifying the molecular changes associated with SNL, we were interested to see if GEG200 and GEG600 could reverse these changes. GEG200 and GEG600 induced changes in the expression of 16 genes (Figure 3B) and 11 genes (Figure 3C), respectively. Relative to the vehicle-treated SNL group, GEG at a 200 mg/kg BW dosage led to an increase in the expression levels of 10 genes (namely, *Slc17a6*, *Chek2*, *Bok*, *Hadc2*, *Birc5*, *Rtn4rl1*, *E2f1*, *Shank3*, *Ldha*, and *Trpm4*) and downregulation of the expression levels of 6 genes (namely, *Hspb1*, *Gadd45g*, *Top2a*, *Fgd2*, *Fos*, and *Nthl1*) in the amygdala of SNL-operated animals (Figure 3B). Compared to the vehicle-treated SNL group, GEG at a 600 mg/kg BW dosage increased the expression level of 1 gene (i.e., *Hadc2*) and decreased the expression levels of 10 genes (i.e., *Fos*, *Gadd45g*, *Cotl1*, *Ncf1*, *Cd68*, *Gdpd2*, *Nthl1*, *Hidac4*, *Bag3*, and *Arc*) in the amygdala of SNL-treated animals (Figure 3C).

**Figure 4:**
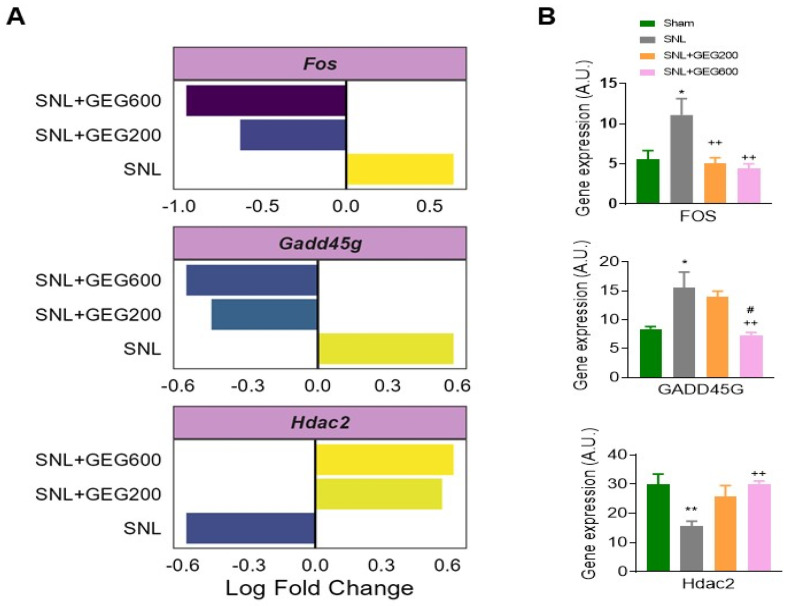
Gene expression analysis of selected neuroinflammation factors, i.e., *Fos*, *Gadd45g*, and *Hdac2* between groups (**A**); mRNA expression assessed by qRT-PCR (**B**). (**A**) Genes show differential expression between all groups, i.e., *p*-value < 0.05 and fold change > 1.25. Log2-fold change in gene expression is shown along the x-axis. SNL indicates fold change between SNL vs. sham, SNL + GEG200 indicates fold change between SNL + GEG200 vs. SNL, and SNL + GEG600 indicates fold change between SNL + GEG600 vs. SNL. (**B**) *Fos*, *Gadd45g*, and *Hdac2* significantly increased in SNL rats (* *p* < 0.05, ** *p* < 0.01 one-way ANOVA, n = 9) compared to sham rats. SNL + GEG200-group rats showed decreased *Fos* expression (++ *p* < 0.01 one-way ANOVA, n = 9), but not *Gadd45g* and *Hdac2* expression, compared to SNL rats. SNL + GEG600-group rats showed decreased *Fos*, *Gadd45g*, and *Hdac2* expression (++ *p* < 0.01 one-way ANOVA, n = 9) compared to SNL rats. SNL + GEG600-group rats showed decreased *Gadd45g* expression (# *p* < 0.05 one-way ANOVA, n = 9) compared to SNL + 200-group rats. Abbreviation: *Fos*, FBJ osteosarcoma oncogene; *Gadd45g*, growth arrest and DNA-damage-inducible 45 gamma; *Hdac2*, histone deacetylase 2.

Next, we focused on the genes of common signatures between the GEG200 group and GEG600 group and those with a dose response associated with GEG concentrations. Relative to the SNL group, both the GEG200 and GEG600 groups showed downregulated *Fos* and *Gadd45g* in the amygdala of SNL rats, as shown in the log fold change of neuroinflammation (Figure 4A) and confirmed with qRT-PCR (Figure 4B). In contrast, both GEG200 and GEG600 groups showed increased *Hdac2* gene expression in amygdala tissue from SNL rats compared to the vehicle-treated SNL group (Figure 4A,B). This suggests that GEG can at least partially reverse the molecular signature in the amygdala associated with neuropathic pain induced by SNL.

### 3.3. GEG-Associated Gut Microbiome Changes

**Figure 5:**
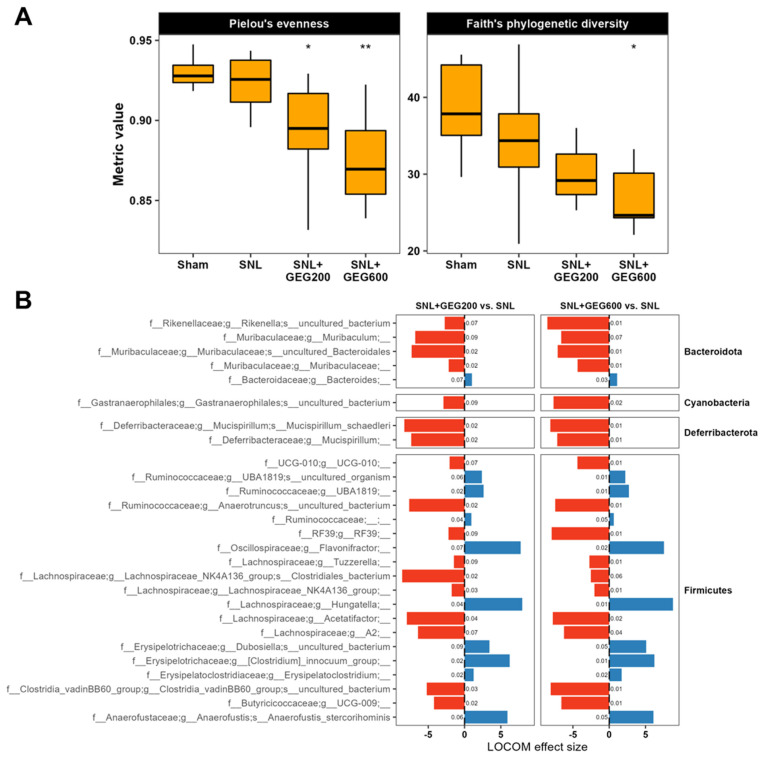
Gut microbiome composition analysis in SNL rats. (**A**) Alpha-diversity evenness and richness are indicated across groups using box plots. Asterisks (* *p* < 0.05, ** *p* < 0.01) indicate statistical significance for SNL + GEG200 vs. SNL and SNL + GEG600 vs. SNL comparisons using the Wilcoxon signed-rank test. (**B**) SNL + GEG200 vs. SNL, SNL + GEG600 vs. SNL compositional microbiome analysis using LOCOM. ASVs presented are those with raw *p*-values < 0.05 and Benjamini–Hochberg-adjusted *p*-values < 0.1. LOCOM effect size is indicated on the x-axis and ASVs are indicated on the y-axis. Benjamini–Hochberg-adjusted *p*-values are indicated next to the corresponding bars.

The effects of GEG supplementation on the gut microbiome of animals are shown in Figure 5. The average sequencing depth per sample was ~524,000 reads. Around 51,000 non-chimeric reads were retained after filtering, denoising, and then merging. First, we examined gut microbiome alpha-diversity. Gut microbiome species (ASV) evenness and richness did not differ between the sham group and the SNL group (Figure 5A). Gut microbiome evenness and richness in GEG-supplemented groups were lower than in the vehicle-treated SNL group. Both the SNL + GEG200 and SNL + GEG600 groups showed significantly lower evenness, but only the SNL + GEG600 group showed significantly lower diversity (Figure 5A) (Wilcoxon signed-rank test, *p* < 0.05).

Next, we aimed to find species associated with SNL and GEG treatments. We performed compositional analysis using LOCOM, a logistic regression model for testing differential abundance in compositional microbiome data with false discovery rate control. Overall, compared to the sham group, the SNL group had only minute changes in the gut microbiome composition and ASV abundance, and none of these changes were statistically significant after false discovery rate control (Benjamini–Hochberg Procedure-adjusted *p*-value > 0.1). Thus, we focused on changes associated with both GEG doses in the SNL groups (SNL + GEG200 vs. SNL and SNL + GEG600 vs. SNL) (Figure 5B) (Benjamini–Hochberg Procedure-adjusted *p*-value < 0.1). GEG supplementation significantly decreased the abundance of ASVs of *f_Rikenellaceae* and *f_Muribaculaceae* in *Bacteroidota phyla*; *g_Gastranaerophilales* in *Cyanobacteria phyla*; and *g_Mucispirilum* in *Deferribacterota phyla* of cecal feces of NP rats. Remarkably, relative to the vehicle-treated SNL group, both the SNL + GEG200 and SNL + GEG600 groups had an increased abundance of 10 ASVs of the taxa in *Firmicutes phyla*, such as *UBA1819*, *Flavonifractor*, *Hungatella*, *Clostridium innocuum group*, *Erysipelatoclostridium*, and *Anaerofustis stercorihominis* (Figure 5B). In contrast, the 17 ASVs of the taxa in *Firmicutes phyla* were decreased in the GEG-supplemented rats compared to the vehicle-treated SNL rats, for instance, *Rikenella*, *Muribaculaceae*, *Gastranaerophilales*, *Clostridia UCG-010*, *Mucispirillum schaedleri*, *RF39*, *Acetatifactor*, *Clostridia*, and *UCG-009* (Figure 5B).

### 3.4. Integrated Analysis of Pain, Neuroinflammatory Markers, and the Gut Microbiome

**Figure 6:**
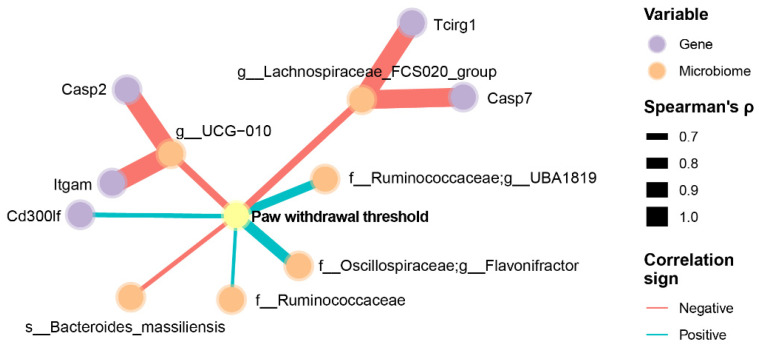
Association between mechanical hypersensitivity, neuroinflammation genes of amygdala, and gut microbiome of cecal feces in GEG-supplemented NP rats. Data were obtained from male SNL rats given GEG at 200 and 600 mg/kg doses daily for 4 weeks. The pain withdrawal threshold was assessed by the von Frey Test. The neuroinflammation gene profile in the amygdala was assessed using the NanoString Neuroinflammation Panel. Gut microbiome composition was assessed by 16S rRNA amplicon sequencing. We employed a network analysis approach with a focus on identifying the genes and microbiota ASVs that are strongly associated with pain withdrawal threshold, determined by Spearman’s rank correlation coefficient >0.6 and *p* < 0.01. Only nodes with edges linked to pain sensitivity were retained in the network. Line thicknesses indicate the strength of the correlation.

We addressed whether NP sensitivity (mechanical hypersensitivity, VFT measurement) is associated with neuroinflammation-related genes and gut microbiome species. Because of the complexity of the collected data and identifying potential mechanisms involving the microbiome–gut–brain axis, we employed a network analysis approach based on Spearman’s correlation coefficient between all three factors, with a focus on identifying the genes and microbiome ASVs that are strongly associated with mechanical hypersensitivity (Spearman’s rank correlation coefficient > 0.6 and *p* < 0.01). Figure 6 shows the exploratory results of network analysis of the correlated paw withdrawal threshold (pain mechanical hypersensitivity), neuroinflammation genes, and gut microbiome ASVs in animals. For example, we found that a higher paw withdrawal threshold (less mechanical hypersensitivity) was positively correlated with the expression of *Cd300lf* and the abundance of *Ruminococcaceae_UBA1819* and *Flavonifractor* (Figure 6). Moreover, a higher paw withdrawal threshold (less mechanical hypersensitivity) was negatively correlated with the abundance of *UGC-010*, *Lachnospiraceae_FCS020_group*, and *Bacteroides massiliensis* (Figure 6).

## 4. Discussion

In the present study, the SNL-induced NP model was successfully employed to examine the effects of two GEG dosages on mechanical hypersensitivity, neuroinflammation/neuroimmune signature genes in the amygdala, and gut microbiome composition in male rats. Both GEG dosages via oral gavage attenuated mechanical hypersensitivity in the SNL-operated animals, independent of GEG doses, which agrees with our previous study with one GEG dosage through dietary supplementation [17,18]. The lack of GEG dose response in this study is also consistent with a previous study showing that the rhizome of *Zingiber officinale* roscoe (*Z. officinale*, ginger) at 100 and 500 mg/kg p.o. mitigated oxaliplatin-treated mechanical allodynia in mice, regardless of *Zingiber officinale* dosages [39]. This study shows for the first time that dietary administration of GEG modulates the neuroinflammation signature genes of the amygdala and gut microbiome composition of male rats with NP, though dose effects of GEG on gut microbiome composition are limited. The data on neuroinflammation genes and the gut microbiome provide strong evidence for the pain-mitigating effects of GEG supplementation in animals with NP through the modulation of the microbiota–CNS connection.

The current study compared, for the first time, gene expression profiles in the amygdala between SNL animals with and without GEG administration and identified two (*Fos* and *Gadd45g*) of three co-expression genes mainly involved in the mitogen-activated protein kinase (*MAPK*) signaling pathway [40]. Fos, an immediate early gene, is considered a general neuronal activity marker, a cause of pain-related increases in neuronal activity. The present study found Fos upregulation in the amygdala of SNL rats, which is consistent with previous work showing *c-FOS* gene and protein upregulation in brain regions in different NP animal models [41,42,43], as well as in the amygdala after spinal neural transection [42]. The change in *c-Fos* after spinal nerve injury triggers the production of *TNF-α* in the anterior cingulate cortex [44]; however, it remains to be determined whether this signaling mechanism is also engaged in the amygdala to contribute to mechanical hypersensitivity during the development of NP. In the present study, supplementation of GEG suppressed the gene expression of Fos in the amygdala, providing an explanation of how GEG mitigates mechanical hypersensitivity in NP.

The current study agrees with Li’s, which showed the upregulation of *Gadd45A* in an SNL model [45]. *Gadd45g* belongs to the *Gadd45* family, which is known to be induced by a myriad of physiological stresses, including irradiation, ultraviolet radiation, and inflammatory cytokines [46,47,48]. The protein encoded by the *Gadd45* gene responds to environmental/physiological stresses by mediating the activation of the *p38/JNK* pathway via MTK1/MEKK4 kinase [49,50]. *Gadd45g* genes are associated with neural cell injury and death, and *Gadd45g* is associated with core promoter lesions hypermethylated in NP development [51]. *Gadd45g* participates in activity-induced neurogenesis by decreasing site-specific DNA methylation in the brain [52,53]. On the other hand, *Gadd45g* interacts with and inhibits the kinase activity of the *Cdk1/CyclinB1* complex [54], suggesting an important role in the progression from the G2 to M phase of the cell cycle [55]. In the present study, our findings of elevated *Gadd45g* expression in the amygdala of SNL rats agree with increased *Gadd45β* in the spinal dorsal horn after SNL surgery in rats [56], in the anterior cingulate cortex after sciatic nerve injury in rats [57], and increased *Gadd45g* protein expression in human nucleus pulposus cells isolated from advanced stages of intervertebral disc degeneration (a form of NP) [50]. In the present study, administration of GEG suppressed the gene expression of *Gadd45g* in the amygdala, suggesting a molecular mechanism by which GEG mitigates mechanical hypersensitivity in NP.

*Hdac2* is involved in transcriptional regulation, cell cycle progression, and chronic NP development [58,59]. The reported changes in *Hdac2* expression are very complex and controversial in different pain models, including arthritis pain [60], neuropathic pain [61,62,63,64,65,66], visceral pain [67,68,69], and bone cancer pain [38,70]. We found that lower *Hdac2* expression in the amygdala of SNL rats agrees with previous finding that SNL procedures diminish *Hdac2* occupancy in the dorsal root ganglion (DRG) of rats [59]. Excess α2δ-1 proteins produced after SNL injury directly interact with glutamate NMDA receptors to intensify synaptic NMDA receptor activity in the spinal cord, a prominent component of nerve discomfort. Because α2δ-1 upregulation after nerve damage is long lasting, gabapentinoids only temporarily relieve pain symptoms. Then, *Hdac2* functions as a pivotal transcriptional repressor of NP via suppressing *Cacna2d1* promoter expression in the DRG [59]. *Hdac2* knockdown or conditional knockout in DRG neurons in male and female mice regularly induced long-lasting mechanical pain hypersensitivity. In the present study, we reported that GEG-supplemented SNL rats had elevated *Hdac2* expression in the amygdala, further corroborating a previous study that restoring the repressive *Hdac2* function and/or reducing histone acetylation at the α2δ-1 gene promoter in primary sensory neurons could lead to long-term nerve pain relief [59].

The approach of regulating gut microbiomes to affect nervous system function represents a new idea for the treatment of NP using bioactive compounds, such as GEG. Accumulating evidence from published work by our group and others [17,61,71] may indicate the gut microbiota’s impact on NP. Our findings show that “potential pro-inflammatory” taxa, such as *Rikenellaceae* [72], *Muribaculaceae* [73], *Mucispirillum* [74], *Rikenella* [75], *Gastranaerophilales* [76], *RF39* [77], *Acetatifactor* [78], and *UGC-009* [79], are decreased in the GEG-supplemented NP rats and support the anti-inflammatory function of GEG in pain mitigation via the modification of gut microbiome composition [74]. In the current study, GEG-treated animals had an increased abundance of *Anaerofustis stercorihominis* and *Hungatella* in cecum feces due to their anti-inflammatory potential. *Anaerofustis stercorihominis* has effects for treating or preventing inflammation-related diseases, such as inflammatory bowel diseases (ulcerative enteritis, gastritis, and general enteritis) and rheumatoid arthritis (WIPO). The dietary inflammatory index is inversely correlated with the relative abundance of the *Hungatella group* [80].

The relationship between gut microbiota and signature gene expression in the amygdala due to GEG supplementation in the context of NP is likely complex. The gut microbiome participates in the metabolism of GEG, and its modulation by GEG supplementation could influence the activity of neuroinflammation genes in the amygdala, resulting in a reduction in mechanical hypersensitivity in animals with NP status. The combination of 16S rRNA gene sequencing and signature neuroinflammation gene analysis in the amygdala can overcome the limitations of single omics to a certain extent. In the present study, the fact that a decreased hypersensitivity level was associated with increased *Cd300lf* gene expression in rats with chronic NP may result from its impeding role in neuroinflammation [81] and may have beneficial effects on amygdala activity in NP. Keswani et al. reported that CD300f, belonging to a family of Ig-like-encoding genes, is a potential candidate associated with cerebral malaria resistance, and the expression of *CD300lf* by microglia strengthens resistance to cerebral malaria by impeding neuroinflammation [81].

This study showed that increased abundance of *Ruminoccaceae_UBA1819* and *Flavonifractor* is associated with reduced hypersensitivity in GEG-supplemented NP rats, in part, due to GEG’s anti-inflammatory response to increased *Ruminoccaceae* [82] and *Flavonifractor* [83]. Furthermore, lessened hypersensitivity was accompanied by a decreased abundance of *Lachnospiraceae_FCS020_group* in the NP rats, which is linked to impaired glucose metabolism and inflammation in type 1 diabetes [84] and may be due to a reduced inflammatory response thanks to GEG. Less hypersensitivity was also negatively associated with the abundance of *Bacteroides massiliensis* in GEG-supplemented NP rats. *Bacteroides massiliensis* was negatively correlated with IL-23 in rats with ulcerative colitis [85]. *IL-23* is a member of the IL-12 family of cytokines with pro-inflammatory properties [86], suggesting that the beneficial effects of GEG involve mitigation of the pro-inflammatory potential of *Bacteroides massiliensis* [85]. The main metabolites of GEG are glucuronide or sulfate conjugates, which may indirectly interact with the gut microbiome of NP rats [87]. Glucuronide conjugates (the metabolites of GEG) likely provide a significant energy source to mammalian GI microbiomes to deactivate endobiotic and xenobiotic compounds for GI excretion [87]. In the GI tract, the microbiota prepares β-glucuronidase enzymes, which remove glucuronic acid as a carbon source, effectively reversing the actions of mammalian intestinal dysbiosis [88]. In the present study, SNL-induced dysbiosis was reversed by GEG supplementation in NP rats, likely due to the action of GEG metabolites.

We noted that this study only used males, and future studies are needed to explore if similar gut–brain interactions and GEG effects are observed in females. The present study provides the rationale for these important but more complex experiments. How the microbiome affects pain pathways is an important knowledge gap. Our study provides the basis and rationale for more research in this area to decipher underlying mechanisms and pathways. While we hypothesize that the gut microbiota plays a role in modulating pain sensitivity, this is most likely to be partial and does not fully explain the complex change in pain sensitivity as other host factors contribute significantly to the phenotype. A contribution of microbiota cannot be ruled out even though there was no detectable change because microbiota could have different functional consequences in the changed environment of the chronic pain condition. Disentangling this complex relationship is very interesting, and we hope the present study will stimulate that line of research. Incorporating cause-and-effect experiments in future studies would significantly enhance the impact of the results reported here. Furthermore, in this study, while it is reasonable to emphasize the investigation of genes (namely, *Fos*, *Gadd45g*, *Hdac2*) shared among distinct groups with connections between neuroimmune signaling and ginger dose response, future studies are warranted to broaden the spectrum to the other genes presented in Figure 3B, 3C that were influenced by ginger. Exploring these genes may provide further insight into the mechanisms of action of GEG.

## 5. Conclusions

Administration of GEG dosages to neuropathic (SNL model) animals decreased mechanical hypersensitivity (no GEG-dose response) and modified the gut microbiome composition (limited GEG dose response). GEG reversed SNL-induced signature genes of neuroinflammation with differential dose response. The data suggest the prebiotic potential of dietary ginger root intake in the management of NP.

## Figures and Tables

**Table 1:** Primer sequences.

Gene	Forward	Reverse
*FOS*	5′-ATC GGC AGA AGG GGC AAA GT-3′	5′-TCC TCC GAT TCC GGC ACT TG-3′
*Gadd45g*	5′-AGT CCG TGG CCA GGA TAC AG-3′	5′-TTT GGC GGA CTC GTA GAC GC-3′
*HDAC2*	5′-GCA CCA CGC CAA GAA GTC AG-3′	5′-ACG GTC ATC ACG CGA TCT GT-3′
*β-actin*	5′-ACA ACC TTC TTG CAG CTC CTC C-3′	5′-TGA CCC ATA CCC ACC ATC ACA-3′

Abbreviations: *FOS*: Fos proto-oncogene, AP-1 transcription factor; *Gadd45g*: growth arrest and DNA-damage-inducible 45 gamma; *HDAC2*: histone deacetylase 2.

## Data Availability

The data presented in this study are available on request from the corresponding author.
